# Bioactive Glasses Containing Strontium or Magnesium Ions to Enhance the Biological Response in Bone Regeneration

**DOI:** 10.3390/nano13192717

**Published:** 2023-10-06

**Authors:** Sílvia Rodrigues Gavinho, Ana Sofia Pádua, Laura Isabel Vilas Holz, Isabel Sá-Nogueira, Jorge Carvalho Silva, João Paulo Borges, Manuel Almeida Valente, Manuel Pedro Fernandes Graça

**Affiliations:** 1 I3N and Physics Department, Aveiro University, 3810-193 Aveiro, Portugal; silviagavinho@ua.pt (S.R.G.); mpfg@ua.pt (M.P.F.G.); 2 I3N-CENIMAT and Physics Department, NOVA School of Science and Technology, Campus de Caparica, 2829-516 Caparica, Portugal; as.padua@campus.fct.unl.pt (A.S.P.); jcs@fct.unl.pt (J.C.S.); 3 TEMA and Mechanical Engineering Department, Aveiro University, 3810-193 Aveiro, Portugal; lauraholz@ua.pt; 4 Associate Laboratory i4HB, Institute for Health and Bioeconomy, NOVA School of Science and Technology, NOVA University Lisbon, 2819-516 Caparica, Portugal; isn@fct.unl.pt; 5 UCIBIO—Applied Molecular Biosciences Unit, Department of Life Sciences, NOVA School of Science and Technology, NOVA University Lisbon, 2819-516 Caparica, Portugal; 6 I3N-CENIMAT and Materials Science Department, NOVA School of Science and Technology, Campus de Caparica, 2829-516 Caparica, Portugal; jpb@fct.unl.pt

**Keywords:** bioactive glass, magnesium, strontium, antibacterial activity, bioactivity, dental applications, implant coatings, bone regeneration, tissue engineering

## Abstract

The non-surgical treatments are being required to reconstruct damaged tissue, prioritizing our body’s natural healing process. Thus, the use of bioactive materials such as bioactive glass has been studied to support the repair and restoration of hard and soft tissue. Thus, in this work Bioglass 45S5 was developed, adding 1 and 2%mol of SrO or MgO and the physical and biological properties were evaluated. The addition of MgO and SrO at the studied concentrations promoted the slight increase in non-bridging oxygens number, observed through the temperature shift in phase transitions to lower values compared to Bioglass 45S5. The insertion of the ions also showed a positive effect on Saos-2 cell viability, decreasing the cytotoxic of Bioglass 45S5. Besides the Ca/P ratio on the pellets surface demonstrating no evidence of higher reactivity between Bioglass 45S5 and Bioglass with Sr and Mg, micrographs show that at 24 h the Ca/P rich layer is denser than in Bioglass 45S5 after the contact with simulated body fluid. The samples with Sr and Mg show a higher antibacterial effect compared to Bioglass 45S5. The addition of the studied ions may benefit the biological response of Bioglass 45S5 in dental applications as scaffolds or coatings.

## 1. Introduction

Given the existence of several bone conditions associated with trauma, infections, tumors or others bone diseases (i.e., osteoporosis), the field of tissue engineering has focused a considerable attention on the process of bone regeneration. However, it has been a concern for clinicians and researchers, the regeneration of large bone defects due to the limiting capacity of bone to regenerate. In order to overcome these limitations, research has targeted the development of an appropriate substitute for the growth of new bone tissue [1,2,3]. Nowadays, the biomaterials are emerging as a biocompatible and bioactive solutions to enhance the bone regeneration process. These biomaterials aims to support the regeneration process, degrading itself and being replaced by the new bone tissue [4,5]. Among biomaterials, bioactive glasses have been proposed as the main strategy in the development of scaffolds, bone cements or as implant coatings for the repair of bone defects or to replace damaged tissues [6,7]. The main advantage of these glasses is the ability to rapidly form a strong bond with the host bone tissue by creating a hydroxyapatite (HA) layer at the interface between bioactive glasses and biological fluids [8,9].

Bioglass 45S5, defined by its composition of 45% SiO_2_, 24.5% CaO, 24.5% Na_2_O and 6% P_2_O_5_ (wt%), presents itself as one of the most widely used inorganic materials for hard tissue regeneration because of its high bioactivity and its bond with the host bone is so strong that it could only be separated by bone fracture. This bioactive glass was developed by Larry Hench through the melt-quenching method in 1969 [10,11]. Besides promoting osseointegration (direct connection between the biomaterial and the host bone), Bioglass 45S5 presents osteoinductive and osteoconductive properties. Osteoinduction allows for the direct induction of bone growth through the recruitment, proliferation and differentiation of mesenchymal stem cells into osteoblasts. Osteoconduction is related to the capacity of the material to act as a matrix and provide the microenvironment to allow adhesion, migration, growth and division of bone cells [4,12,13,14,15].

The biological behavior of the Bioglass 45S5 can be enhanced with the addition of bivalent therapeutic ions such as strontium, magnesium, etc. This is mainly due to the presence of these ions in the natural composition of hard tissues and their role in bone growth and regeneration. Strontium ion plays an important function in the process of bone remodeling, promoting the proliferation and differentiation of pre-osteoblastic cells into osteoblasts while inhibiting osteoclastic differentiation and activity. It has been observed that Sr^2+^ increases alkaline phosphatase (ALP) activity and the expression of osteoblast-related genes, is non-toxic to cells, even at high concentrations, can increase the capacity for apatite development by decreasing the time of its formation, and can promote bone densification [16,17,18,19,20,21]. Magnesium ion is the fourth most abundant ion in the body and 67% is stored in bones and teeth. Thus, Mg^2+^ can promote new bone formation, stimulating proliferation and differentiation of osteoblasts. This stimulus is based on influence of magnesium at different signaling pathways and direct interaction with integrins, present in osteoblasts, which are responsible for cell adhesion and stability. It also increases the ability of the bioactive glass to form the apatite layer during in vivo implantation [22,23,24,25,26]. In addition, some studies reported that the insertion of these ions to the network of Bioglass 45S5 could enhance its antibacterial effect [3,15,17,27]. Both ions have been studied and used in soft and hard tissue reconstruction in the maxillofacial region. Magnesium and strontium ions have been added to biodegradable structures for soft tissue regeneration, in enamel repair by inducing differentiation and proliferation of human dental pulp cells, as surface treatment of dental implants to improve the integration of gingival epithelial cells and fibroblasts, and have antibacterial effect for common periodontal pathogens. Some magnesium-based materials promote beneficial mechanical properties with elastic modulus similar to bone and on the other hand, the addition of strontium shows interesting results in the prevention of demineralized lesion formation and on prevention of artificial dentine caries if contained in toothpaste [28,29,30,31,32,33,34,35].

The main goal of this work is to develop a material that enhance the formation of new bone in shorter times to favour the bonding of the material to the host bone and to avoid the formation of a biofilm. Thus, to improve this connection, bioactive glasses with Sr^2+^ or with Mg^2+^ were developed. The bioactive glasses (BG) were synthesized with the formulation proposed by L. Hench, adding SrO (1 and 2 mol%, BGSr1 and BGSr2) or MgO (1 and 2 mol%, BGMg1 and BGMg2). The samples were thermal and structurally characterized and their biological performance was evaluated to investigate the effect of the insertion of SrO and MgO in Bioglass 45S5 on cytotoxicity, bioactivity and antibacterial activity.

## 2. Materials and Methods

### 2.1. Materials Synthesis

The bioglass (BG) was fabricated using the melt-quenching method taking into account the Bioglass 45S5 composition reported by Hench et al. [11,36]. Several concentrations of MgO (1 and 2 mol%) and SrO (1 and 2 mol%) were added to the bioactive glass network (Table 1). All compositions (BG, BGSr1, BGSr1, BGMg1 and BGMg2) were obtained by mixing for 1 h at 300 rpm by planetary ball milling process. The starting chemicals were SiO_2_, P_2_O_5_, CaCO_3_, Na_2_CO_3_ and MgO or Sr(NO_3_)_2_ (all reagents were supplied by Sigma-Aldrich, Germany, high purity). The mixed reagents were heat treated for 8 h at 800 °C (Termolab furnace, Portugal) to remove the NO_3_ and CO_3_ of the initial materials.

The melting process was performed at 1300 °C for 1 h in a platinum crucible. The bioactive glass was re-melted under the same parameters to improve the homogeneity of the samples. The bulk material resulting from the melt-quenching was crushed in order to decrease the particle size and the particle size distribution. The powder resulting from the manual grinding process was milled in a planetary ball mill system (PULVERISETTE 7 from Fritsch, Germany) for 60 min at 300 rpm, using 25 agata balls of 10 mm in diameter in each of the vessels.

### 2.2. Thermal Analysis

Differential thermal analysis (DTA) and thermogravimetric analysis (TG) were carried out on a Hitachi STA 7300 from room temperature up to 1200 °C with a heating rate of 5 °C/min. The DTA measurements were performed in BGSr2 and BGMg2 powder samples obtained from the ball milling process, described in the previous procedure, under a 200 mL/min Nitrogen N50 (99.999%) atmosphere. The measurements were platinum crucibles were used. The glass transition (T_g_) was calculated by analyzing the extrapolated tangents methodology.

### 2.3. Structural Characterization

The structure of the bioactive glass powder was characterized by XRD (X-ray powder diffraction) and FTIR (Fourier-transform infrared spectroscopy).

#### 2.3.1. XRD

The XRD diffractograms were obtained at 25 °C on an Aeris-Panalytical diffractometer. CuKα radiation (λ = 1.54056 Å) was generated with 40 kV and 15 mA. The samples were assessed with a 2θ range of 10° up to 70° and a scan step of 0.002°. For grazing incidence measurements (GIXRD), a Rigaku SmartLab diffractometer (CuKα radiation—1.5406 Å, 40 kV, 30 mA) was used. The patterns were obtained in the range of 10° < 2θ < 70°, scan speed of 0.6° min^−^^1^ and with an incident angle of 5°.

#### 2.3.2. FTIR

FTIR spectroscopy was assessed with a Perkin-Elmer Spectrum BX FTIR™ spectrometer from 1200 cm^−1^ to 400 cm^−1^ with a resolution of 4 cm^−1^ and 128 co-added scans. The data acquisition was performed at room temperature (approximately 25 °C) and with 37% of humidity.

### 2.4. Cytotoxicity Assay

The cytotoxicity of all samples towards Saos-2 (human osteosarcoma cell line ATCC^®^ HTB-85™) was performed according to the “ISO 10993-5 Biological evaluation of medical devices—Part 5: Tests for in vitro cytotoxicity” standard, using the extract method. The sterilization process of the powders was performed at 120 °C for 2 h. The BG powder was incubated in McCoy’s 5A medium for 24 h at 37 °C, resulting in: (i) a non-passivated extract; (ii) a passivated bioactive glass powder. To obtain a passivated extract, the passivated bioactive glass powder was again incubated in McCoy’s 5A medium at 37 °C for 24 h. Both extracts, passivated and non-passivated, were produced at a concentration of 100 mg/mL and filtered with a 0.22 µm cellulose acetate filter and stored at 37 °C [37].

The cells were seeded in 96-well plates and incubated for 24 h at 37 °C in a humidified atmosphere with 5% CO_2_. The negative control consisted of viable cells, and the positive control consisted of cells cultured in a cytotoxic medium promoted by the supplementation of the medium with 10% dimethyl sulphoxide (DMSO). 24 h after seeding, cells were exposed to both passivated and non-passivated extracts at the highest concentration (100 mg/mL) and to serial dilutions (50 mg/mL, 25 mg/mL and 12.5 mg/mL).

A colourimetric viability assay using resazurin solution (Alfa Aesar, Ward Hill, MA, USA) was performed 48 h after of cell exposure to the extracts. Resazurin is a very low cytotoxic nonfluorescent blue dye that indicates the cell viability. Resazurin is reduced by live cells to resorufin, a pink fluorescent compound. The resazurin solution and culture medium in a *v*/*v* ratio of 1:1 were incubated for 3 h and the absorption was measured as described in previous work [38].

Three independent replicates were performed with six statistical replicates.

The cytotoxicity or non-cytotoxicity of the extracts were discussed according to the classification in the Table 2. 

### 2.5. Bioactivity

The bioactivity assay was performed following “Implants for surgery—In vitro evaluation for apatite-forming ability of implant materials” (ISO 23317:2014) and as stablished by Kokubo et al. [39]. Pellets with 7 mm of diameter were immersed in simulated body fluid (SBF), an ionic solution with composition similar to the human plasma (Table 3), for 12 h, 24 h, 48 h, 96 h, 336 h and 672 h. The volume of SBF ($V_{s})$, in mm^3^, placed in contact with the pellets considering their apparent surface area as indicated in the following equation:

(1) $$V_{s}=100\cdot \;S_{a}$$

where $S_{a}$ is the superficial area of the sample. All pellets after immersion were washed with ultrapure water. The sample’s surface was analyzed before and after immersion times with SEM-EDS from TESCAN VEGA 3 (TESCAN, Brno, Czech Republic). A semi-quantitative study of the atomic elements percentage on the surface’s samples was made using the Bruker EDS system coupled to the microscope. In addition, the pH of the SBF medium for the all samples was measured at the end of the immersion times, as previously described [40]. The assay was performed in duplicate.

### 2.6. Antimicrobial Effect

The antimicrobial effect of all the samples were assessed by the method of agar diffusion assay. The pellets with 7 mm of diameter and ~2 mm of thickness were previously sterilized at 180 °C for 2 h. The study was performed against the strains *Staphylococcus aureus* COL MRSA (methicillin-resistant strain, provided by Rockefeller University, New York, NY, USA), *Escherichia coli* K12 DSM498 (DSMZ, Braunschweig, Germany) and *Streptococcus mutans* DSM20523 (DSMZ, Germany). The bacterial strains were incubated, at 37 °C, overnight in TSB (tryptic soy broth). The two-layer bioassay was performed using the TSB solidified, as previously described [41]. The concentration of the bacteria in the top layer was approximately 10^8^ CFU/mL. The pellets were placed on the plates and were incubated for 24 h at 37 °C. For *S. mutans*, an incubator kept at 5% CO_2_ was used [40]. As negative and positive controls sterile paper discs of 6 mm diameter (FILTER-LAB^®^) impregnated with bi-distilled water and gentamicin 10 µg, respectively, were used in the agar diffusion assay, as described in Figure S1.

Photographs of the pellets were taken, and the diameters of the inhibition halos were measured, 50 times in several directions, using ImageJ software (USA) [42]. The study was performed in three biological replicas. The data were statistically analyzed with an unpaired t-test, comparing the BG composition with each of the different samples (BGSr1, BGSr2, BGMg1 and BGMg2) using GraphPad Prism 8.0 software (USA).

## 3. Results

### 3.1. Differential Thermal Analysis (DTA)

**Figure 1:**
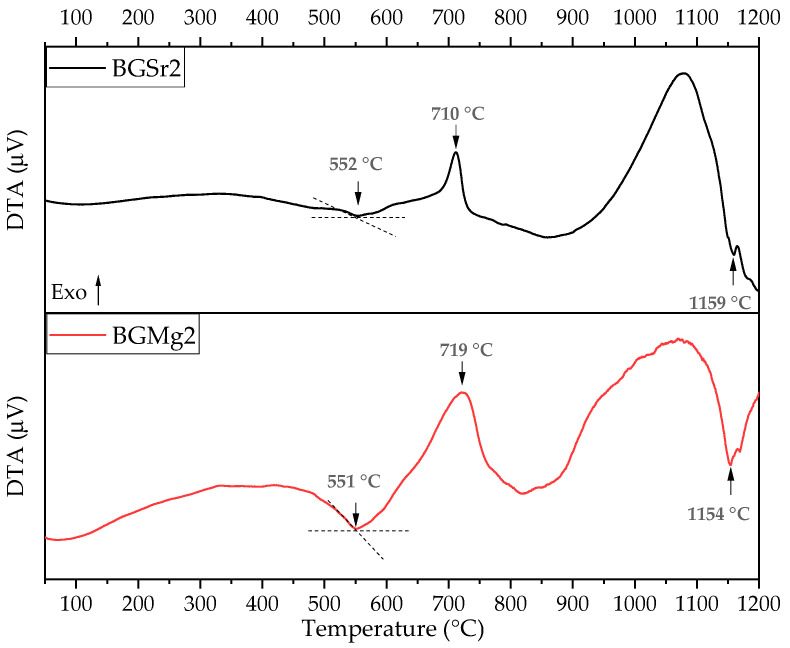
Differential thermal analysis (DTA) curve of BGSr2 and BGMg2 powders.

Figure 1 represents the thermal behavior of the BGSr2 and BGMg2 powders. The thermograms show an endothermic peak around 551 °C and 552 °C for BGSr2 and BGMg2, respectively, a well-defined exothermic phenomenon at 710 °C for BGSr2 and 719 °C for BGMg2, and a second endothermic peak at approximately 1154–1159 °C (Table 4).

### 3.2. Physical Characterization

#### 3.2.1. X-ray Diffraction (XRD)

**Figure 2:**
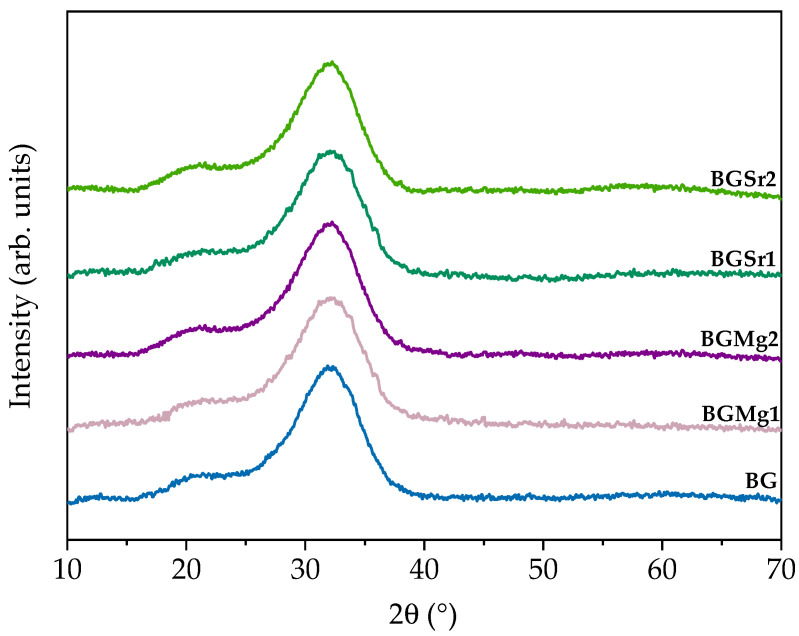
X-ray diffraction patterns for the bioactive glass sample and for BGSr1, BGSr2, BGMg1 and BGMg2.

The XRD diffractograms presented in Figure 2 show a broad band at 2θ = ~26–37°, in all samples. The addition of strontium and magnesium did not promote any phase transition, even in the samples with the highest concentrations.

#### 3.2.2. Fourier Transform Infrared Spectroscopy (FTIR)

**Figure 3:**
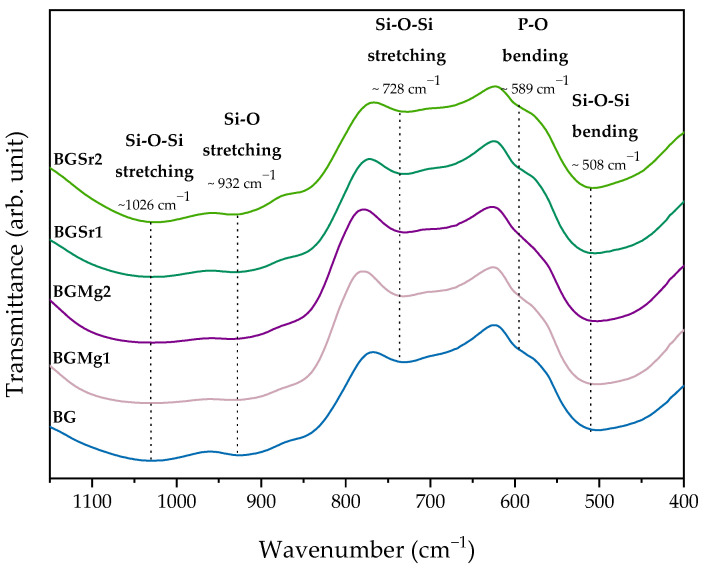
FTIR spectra of BG, BGMg1, BGMg2, BGMSr1 and BGSr2. The relevant vibrations are identified by the corresponding wavenumber.

The FTIR spectra shown in Figure 3 present the same vibration bands in all samples. The addition of both bivalent cations to the Bioglass 45S5 network did not affect the typical vibration bonds. The vibration bands characteristic of this type of bioactive glass is identified in the spectrum. The bands at 1026 cm^−1^and 728 cm^−1^ are related to Si-O-Si stretching mode and 932 cm^−1^ is assigned to Si-O stretching mode. The bending mode at 589 cm^−1^ and 508 cm^−1^ are associated with P-O and Si-O-Si, respectively.

### 3.3. Biological Behaviour

#### 3.3.1. Cytotoxicity

**Figure 4:**
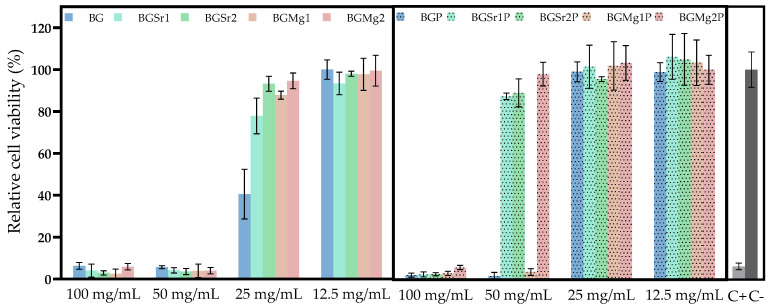
Cell viability of osteosarcoma cell line (Saos-2) after 48 h incubation with non-passivated (BG, BGSr1, BgSr2, BGMg1 and BGMg2) and passivated (BGP, BGSr1P, BGSr2P, BGMg1P and BGMg2P) bioactive glass extracts. Dashed line is related to the percentage of cell viability above which extracts are non-cytotoxic.

Figure 4 shows the results of the cytotoxicity assay on the Saos-2 cell line, by the extract method, for non-passivated and passivated samples. The extracts from non-passivated samples show cytotoxicity for all samples up to 50 mg/mL. At the concentration of 25 mg/mL, the samples with Sr and Mg ions show cell viability above 80% while the base still shows cytotoxicity. Extracts of the passivated samples BGSr1, BGSr2 and BGMg2 stop showing cytotoxicity at the concentration of 50 mg/mL. The base and the sample with 1% Mg still show toxicity for Saos-2 cells. However, at 25 mg/mL all samples are no longer cytotoxic.

#### 3.3.2. Bioactivity

**Figure 5:**
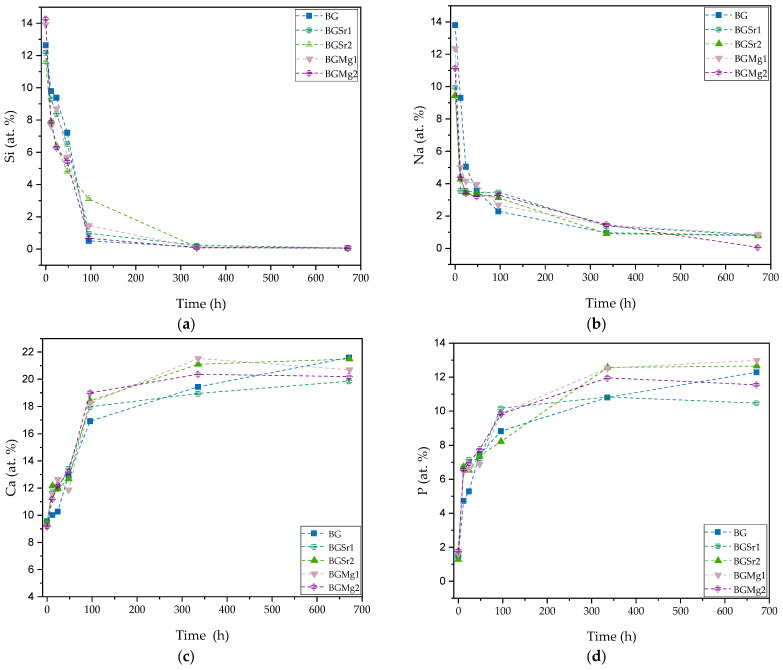

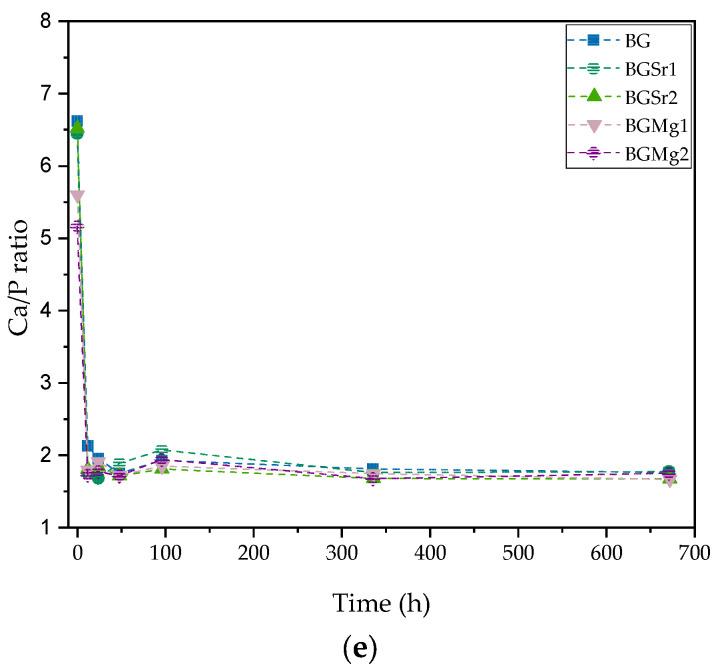
Atomic percentage of the ions presented on the surface of the pellets after SBF immersion for 12 h, 24 h, 48 h, 96 h, 336 h and 672 h. (**a**) silicon at.%; (**b**) sodium at.%; (**c**) calcium at.%; (**d**) phosphorous at.% and (**e**) ratio between calcium and phosphorous.

**Figure 6:**
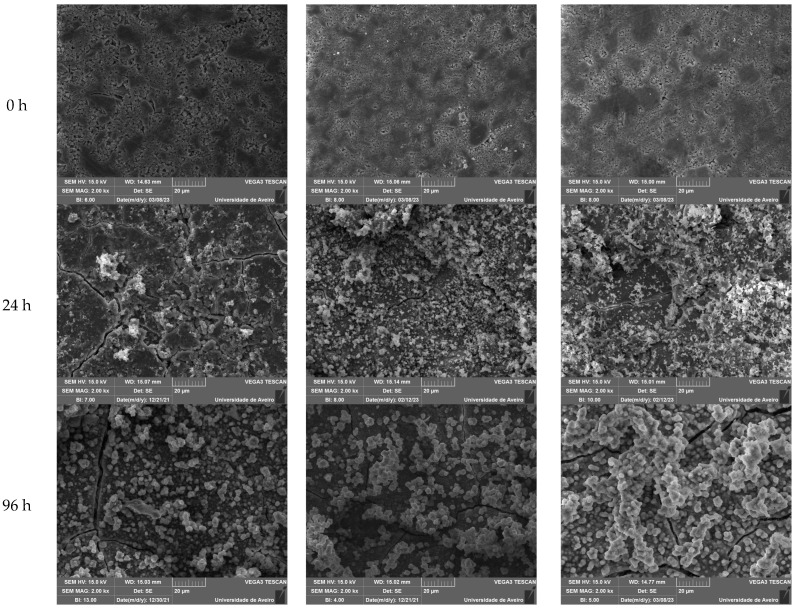

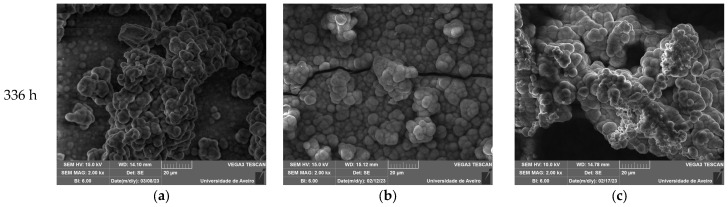
SEM image of the surface of the pellets BG (**a**) BGSr2 (**b**) and BGMg2 (**c**) before and after SBF immersion for 24 h, 96 h and 336 h.

**Figure 7:**
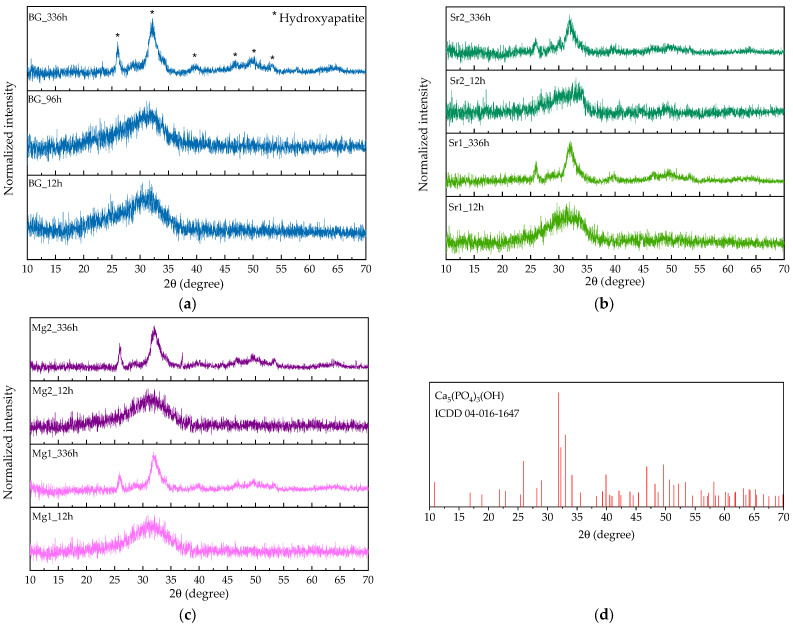
GIXRD spectra of BG (**a**) BGSr (**b**) and BGMg (**c**) samples after SBF immersion for 12 h, 96 h and 336 h; (**d**) XRD pattern to the peak list of Ca_5_(PO_4_)_3_(OH) (* Hydroxyapatite).

In order to evaluate the reaction kinetics in physiological environment regarding the precipitation of apatite on the surface of the bioactive glass, the pellet materials were immersed in simulated body fluid (SBF) for 12 h, 24 h, 48 h, 96 h, 336 h and 672 h. The atomic elements were analyzed by SEM-EDS in relative percentage. Analyzing the graphs of Figure 5a,b it is evident the decrease of silicon and sodium ions at the surface of the bioactive glass over time, stabilizing after 96 h. Otherwise, it is observed the increase of calcium and phosphorus at the surface of the pellets is represented in Figure 5c,d. Figure 5e presents the ratio between calcium and phosphorus (Ca/P) showing values close to 1.67 for samples with SrO and MgO from 12 h in SBF medium. For the studied immersion times, the Sr and Mg bioglasses do not present evident differences when compared to BG, in the Ca/P ratio values. However, in micrographs, the samples immersed for 24 h (Figure 6, line 2), it is visible a deposition of particles related with apatite layer in a higher amount for the BGSr and BGMg samples when compared to BG sample. The micrographs show an increase in the number of particles deposited on the surface and the increase in their size over the immersion time (Table 5). Regarding particle diameter, their values increase from 200–300 nm, at 24 h, to approximately 6 µm, at 336 h of SBF immersion for all samples. Figure 7 shows the grazing XRD measurements of the BG, BGSr and BGMg samples after SBF immersion for 12 h, 96 h and 336 h. The patterns of the samples immersed for 336 h present XRD diffraction peaks.

**Figure 8:**
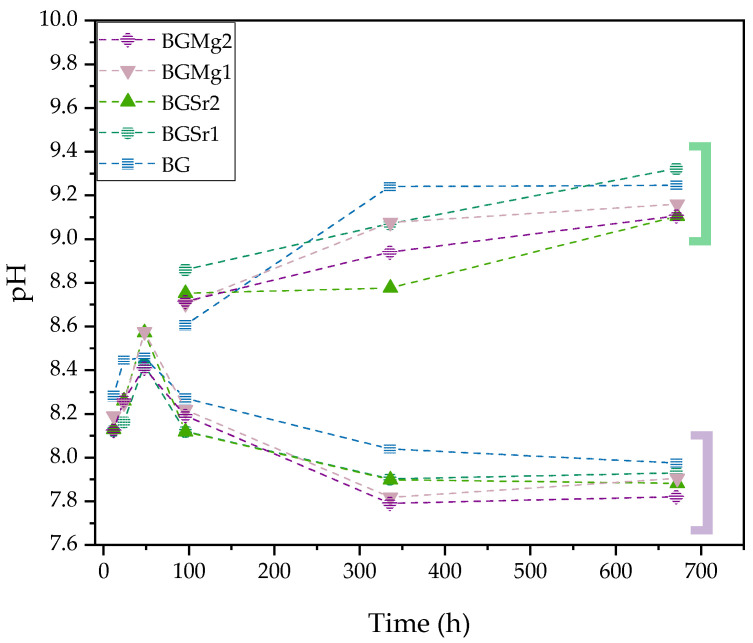
pH values of the SBF resulting of the pellets immersion (BG, BGSr1, BGSr2, BGMg1 and BGMg2) for 12 h, 24 h, 48 h, 96 h, 336 h and 672 h. Green parenthesis: Extracts pH without SBF medium change; Purple parenthesis: Extracts pH with SBF medium change.

Figure 8 shows the graph of the pH values of SBF after several immersion times (12 h, 24 h, 48 h, 96 h, 336 h and 672 h) of the pellets with changing of the medium every two days and without changing the medium. The pH increased on samples without changing SBF medium (green parenthesis).

#### 3.3.3. Antimicrobial Activity

**Figure 9:**
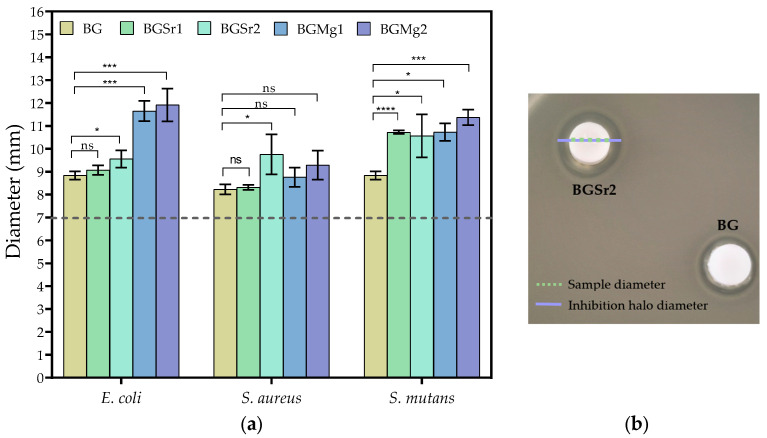
(**a**) Values of the inhibition halo diameters of all samples against *E. coli*, *S. aureus* and *S. mutans* bacteria after incubation for 24 h (statistical analysis was performed using unpaired t-test and the *p*-values indicate the statistical significance; ns: non significant; * *p* ≤ 0.05; ** *p* ≤ 0.01; *** *p* ≤ 0.001; **** *p* ≤ 0.0001); (**b**) Representative photograph of the antibacterial assay result against *E. coli* for BGSr2 and BG samples with 7 mm of diameter represented by the dashed line.

The antimicrobial activity was tested by the agar diffusion method against the bacteria *E. coli*, *S. aureus* and *S. mutans*. Figure 9 shows the values of the diameters of the inhibition halos of the pellets for several bacteria. 

For *E. coli*, the highest antimicrobial effect was observed in the samples with added magnesium. In the strontium samples, only the sample with 2 mol% showed significant antimicrobial activity concerning the Bioglass 45S5 sample. For the Gram+ bacteria *S. aureus*, the antimicrobial effect is not very evident in all samples compared to the BG, with the greatest antimicrobial effect being observed in sample BGSr1. The inhibition halos observed against *S. mutans* show that all samples with added Sr and Mg ions present a higher antimicrobial effect than the BG.

## 4. Discussion

Figure 1 shows the thermal behaviour of the bioactive glass 45S5 with 2 mol% of SrO or MgO. The graph shows the main phase transitions of Bioglass 45S5 [26,43]. The first endothermic phenomenon, associated with T_g_, is observed between 551–552 °C and the second one, related to the melting temperature (T_m_), at 1054–1059 °C. The only peak corresponding at exothermic phenomenon associated with the crystallization temperature (T_c_) is demonstrated at 710–719 °C. Analyzing the results, a deviation of the glass transition temperature is visible for both samples. In addition, the crystallization temperature and the melt temperature also show a decrease in their values compared to the bioactive glass 45S5 (Table 1). This deviation towards lower temperature values of the transitions associated with endothermic and exothermic phenomena is related to the expansion of the glass network by the addition of network modifiers ions [44]. This expansion is due to the depolymerization of the network connectivity by increasing the ratio of non-bridging oxygen number (NBO) to bridging oxygen number (BO) bonds [45,46,47]. Thus, the addition of the cationic ions influences the network structure of Bioglass 45S5, weakening it as already reported in other studies [48,49].

The structural properties of all samples were analyzed by XRD and FTIR techniques. Figure 2 shows the XRD patterns and, as expected, the BG confirms the amorphous nature of Bioglass^®^, after the quenching and milling process, by its typical band in the 2θ range between 25–40°. The addition of ions, both in BGSr and BGMg samples, even at the highest molar concentrations, does not modify the BG network. The BGSr and BGMg samples do not present any peak associated with crystalline phases as previously confirmed in other studies [50,51].

The FTIR spectrum shows the absorption bands of all samples (Figure 3). The vibration bands observed in the graph are similar for all samples and do not present addition of new bands with the insertion of Sr or Mg ions at concentrations up to 2 mol%. In all graphs are identified the main bands relative to the amorphous Bioglass^®^. The vibration bands around 1026 cm^−1^ is related to the Si-O-Si asymmetric stretching mode, 932 cm^−1^ is assigned to Si-O stretching and 728 cm^−1^ is associated to the Si-O-Si symmetric stretching mode [48,52]. The vibration band at 508 cm^−1^ is associated to the Si-O-Si bending mode. The vibration band at 589 cm^−1^ is associated with P-O bending mode [53].

The result of the cytotoxicity analysis of the extracts on osteosarcoma cells is shown in Figure 4. Severe cytotoxicity level is visible for all samples in non-passivated extracts up to 50 mg/mL. However, the results for the extracts of the samples with SrO and MgO insertion show cell viability above 80% at 25 mg/mL. The evident cytotoxicity in the results of the non-passivated extracts is due to the first stage reaction between the bioactive glasses and the cell culture medium that promotes a burst release of ions by increasing the pH value of the medium, as shown in Figure 8, and promoting cell death [19]. To mimic the behaviour that exists in the organisms, which always tends to equilibrate the pH, a new cell medium was added to the bioactive glasses passivated, avoiding the first release of the ions [54]. The results from the cell viability assays for the passivated extracts show that the BGSr samples do not present toxicity for Saos-2 at 50 mg/mL. The BG and BGMg1 samples exhibit non-cytotoxic behaviour below 25 mg/mL. The insertion of Sr^2+^ and Mg^2+^ ions, at the tested concentrations, decreases the cytotoxicity level of Bioglass 45S5, which is in agreement with previous studies [51,55]. In addition, and as shown in recent studies, the Sr^2+^ ion can stimulate the proliferation of like-osteoblast cells and bioactive glass with Mg^2+^ shows good biocompatibility, is less harmful to cell viability and promotes a higher cell proliferation when compared to Mg-free bioactive glass [47,51,55,56].

The bioactivity and the dissolution of the glass are evaluated through the formation of the apatite layer that should present a Ca/P ratio close to 1.67. Figure 5 and Figure 6 present the values of the relative amount of the glass cations elements and of the morphology of those glasses’ surfaces, after immersion in SBF, respectively.

The reaction mechanism that leads to the formation of apatite and subsequently to the formation of new bone starts with the instantaneous exchange between the monovalent (Na^+^) and divalent (Ca^2+^) ions present in the glass and the H^+^ ions present in the fluid. The decrease in the amount of Na^+^ on the glass surface is seen in Figure 5b. At 12 h, for samples with the presence of Sr and Mg, this release is more evident, showing more reactivity of the glass. The insertion of these bivalent ions to the bioglass network, at these concentrations, allows the expansion of the network and consequently increases the ionic dissolution rate. The formation of the silica gel layer on the glass surface enhances the diffusion of Ca^2+^ and PO_4_^3−^ ions present in the glass and the absorption of Ca and P ions from the solution, forming an amorphous calcium-phosphate layer. This set of reactions can be identified in Figure 5a,c,d by the decrease in silicon and the increase in calcium and phosphorus detected on the glass surface over the immersion time. The ratio between Ca and P, shown in Figure 5e, reveals a converging trend toward a value of 1.67, which is a characteristic of hydroxyapatite. The calculated Ca/P ratio being close to the expected value (1.67), and not lower, indicates that the formation of a calcium-deficient hydroxyapatite phase (1.5 to 1.67) does not occurs [57].

At the immersion times studied the difference, in such ratio, is not evident for the BG sample and the samples with Sr and Mg. However, at 12 h the BG presents Ca/P = 2.12 and the samples with Sr and Mg present Ca/P ratios between 1.80 and 1.71, values closer to 1.67 when compared with BG. This faster approximation to this ratio, suggests that the insertion of the two ions promoted a slight change in the reactivity of the BG. As shown in DTA results, the insertion the Sr and Mg led to an increase in the number of NBO’s and previous studies have suggested that higher amount of modifiers in the glass network contribute to higher reactivity in the physiological medium [1,18,54,58,59,60,61].

The evolution of the deposition of the hydroxyapatite layer on the surface of the samples after immersion in SBF is shown in Figure 6. For the samples BG, BGSr2 and BGMg2 before and after the immersion at 24 h, 96 h and 336 h. Observing the morphology of the surface it is visible the existence of particles from 24 h and their number increases with the increase of immersion hours, filling the surface of the pellet. At 24 h a higher number of particles is observed in the BGSr2 and BGMg2 samples, indicating the influence of the addition of these ions on the bioactivity of the bioactive glass. The spherical particles tend to increase in diameter and to agglomerate for longer immersion times, reaching values of approximately 6 µm [62]. Figure 7 reveals the formation of a crystalline phase by showing, at 336 h of SBF immersion, the main diffraction peaks of hydroxyapatite [63].

Figure 8 shows the graph of the pH values of SBF after several immersion times of the pellets. One group of pellets went through the test without changing the medium and another group changed the medium every 2 days to mimic the behaviour of the material in the organism [64]. As expected, the incubation of the pellets in SBF showed an increase in pH to values above 9.2, with a sharp rise in the first 4 days. This pH increase above neutral shows the continuous release of alkaline ions in agreement with the reaction mechanism mentioned above and verified by other studies [40,65]. In order to mimic the pH equilibrium maintained by the organism the medium was replaced every two days. This group of samples showed a decrease in the pH, stabilizing the values after 4 days, corroborating the analysis that was verified in the cell viability results in which the first burst release increases the pH and is toxic for the cells and that passivated samples in which there is the addition of new medium decreased the toxicity due to the decrease in pH. The decrease in pH and its stabilization indicates the formation of the hydroxyapatite layer on the surface of the bioactive glass [66].

Figure 9 shows the inhibition halo diameter values comparing BGSr or BGMg samples with BG. For *E. coli*, BGMg1 and BGMg2 samples show a very pronounced antimicrobial effect compared to BG and BGSr samples. All samples tested against *S. aureus* showed an antimicrobial effect, but the insertion of Sr and Mg did not show a greater effect than the base, except for BGSr1. However, against *S. mutans* all samples with Mg and Sr show a superior antimicrobial effect than Bioglass 45S5. These results are in line with studies that revealed that the presence of Mg^2+^ or Sr^2+^ into Bioglass 45S5 network affects bacterial proliferation, inhibiting its growth [3,15,16,17,21,27].

## 5. Conclusions

All the studied glasses (BG, BGSr and BGMg), developed by the fusion method, showed amorphous structure verified by XRD and FTIR, typical of Bioglass 45S5, even for the samples with SrO and MgO addition. The cell viability of BG was improved with the addition of Sr^2+^ and Mg^2+^, showing no cytotoxic effect to the extract concentration up to 25 mg/mL. Although the influence on the bioactivity of BG with the addition of SrO and MgO is not so evident in the Ca/P ratio study, the evaluation of the surface morphology of the samples, at 24 h of immersion, shows a higher percentage of deposition of the apatite layer in the samples with Sr and Mg than in BG. Furthermore, BGSr and BGMg show a significant antimicrobial effect when compared to BG. Thus, the addition of divalent ions to the network of Bioglass 45S5 shows promising results to promote bioactivity and prevent biofilm formation, facilitating the osteointegration of the material when applied in the field of regenerative medicine.

## Figures and Tables

**Table 1:** Molar composition (mol%) of bioactive glasses studied.

Samples	SiO_2_	P_2_O_5_	Na_2_O	CaO	SrO	MgO
BG	46.1	2.6	24.35	26.91	-	-
BGSr1	45.64	2.57	24.11	26.64	1.00	-
BGSr2	45.18	2.55	23.86	26.37	2.00	-
BGMg1	45.64	2.57	24.11	26.64	-	1.00
BGMg2	45.18	2.55	23.86	26.37	-	2.00

**Table 2:** Qualitative morphological grading according to the cytotoxicity of the extracts.

Grade	Reaction	Culture Conditions
0	None	No cell lysis, no reduction of cell growth
1	Slight	Not more than 20 % of the cells loosely attached or show changes in morphology; only slight growth inhibition visible
2	Mild	No extensive cell lysis; not more than 50 % growth inhibition visible
3	Moderate	Cell layers not completely destroyed, but more than 50 % growth inhibition visible
4	Severe	Nearly complete or complete destruction of cell layers

**Table 3:** Ions concentration in SBF vs. human blood plasma.

Ions	Concentration (10^−3^ mol)	
	SBF (ISO 23317:2014)	Human Blood Plasma
${Na}^{+}$	142.0	142.0
${Cl}^{-}$	147.8	103.0
${HCO}_{3}^{-}$	4.2	27.0
$K^{+}$	5.0	5.0
${Mg}^{2+}$	1.5	1.5
${Ca}^{2+}$	2.5	2.5
${HPO}_{4}^{2-}$	1.0	1.0
${SO}_{4}^{2-}$	0.5	0.5

**Table 4:** Values of T_g_, T_c_ and T_m_ of BG, BGSr2 and BGMg2 powders.

	T_g_ (°C)	T_c_ (°C)	T_m_ (°C)
BG [43]	559	728	1175
BGSr2	552	710	1159
BGMg2	551	719	1154

**Table 5:** Mean size of particles (mm) on pellet’s surface after immersion in SBF medium.

Samples	24 h	96 h	336 h
BG	0.27 ± 0.05	2.14 ± 0.25	5.99 ± 1.67
BGSr2	0.20 ± 0.03	2.8 ± 0.33	6.57 ± 0.91
BGMg2	0.37 ± 0.10	2.73 ± 0.64	5.78 ± 0.61

## Supplementary Materials

The following are available online at https://www.mdpi.com/article/10.3390/nano13192717/s1, Figure S1: Disc diffusion method using (a) *Escherichia coli* K12 DSM498; (b) *Staphylococcus aureus* COL MRSA and (c) *Streptococcus mutans* DSM20523. The *S. mutans* strain used in this work displays resistance to 10 µg of gentamicin. Discs impregnated with bi-distilled water (−) and gentamicin (10 µg) (G).
