# Supplementary material for: Bioactive Glasses Containing Strontium or Magnesium Ions to Enhance the Biological Response in Bone Regeneration
Source: Nanomaterials (Basel). 2023 Oct 6;13(19):2717. doi: 10.3390/nano13192717 (PMC10574208; doi:10.3390/nano13192717)
Supplement: Supplementary file 1 [file nanomaterials-13-02717-s001.zip › nanomaterials-2505589-supplementary.pdf]

Supplementary Materials

Bioactive Glasses Containing Strontium or Magnesium Ions to Enhance the Biological Response in Bone Regeneration

Sílvia Rodrigues Gavinho [1], Ana Sofia Pádua [2], Laura Isabel Vilas Holz [3], Isabel Sá-Nogueira [4,5], Jorge Carvalho Silva [2], João Paulo Borges [6], Manuel Almeida Valente [1,*] and Manuel Pedro Fernandes Graça [1]

1 I3N and Physics Department, Aveiro University, 3810-193 Aveiro, Portugal; silviagavinho@ua.pt (S.R.G.); mpfg@ua.pt (M.P.F.G.)
2 I3N-CENIMAT and Physics Department, NOVA School of Science and Technology, Campus de Caparica, 2829-516 Caparica, Portugal; as.padua@campus.fct.unl.pt (A.S.P.); jcs@fct.unl.pt (J.C.S.)
3 TEMA and Mechanical Engineering Department, Aveiro University, 3810-193 Aveiro, Portugal; lauraholz@ua.pt
4 Associate Laboratory i4HB—Institute for Health and Bioeconomy, NOVA School of Science and Technology, NOVA University Lisbon, 2819-516 Caparica, Portugal; isn@fct.unl.pt
5 UCIBIO—Applied Molecular Biosciences Unit, Department of Life Sciences, NOVA School of Science and Technology, NOVA University Lisbon, 2819-516 Caparica, Portugal
6 I3N-CENIMAT and Materials Science Department, NOVA School of Science and Technology, Campus de Caparica, 2829-516 Caparica, Portugal; jpb@fct.unl.pt
* Correspondence: mav@ua.pt

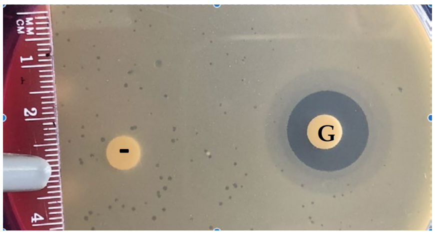

(a)

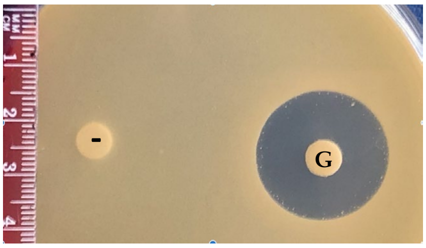

(b)

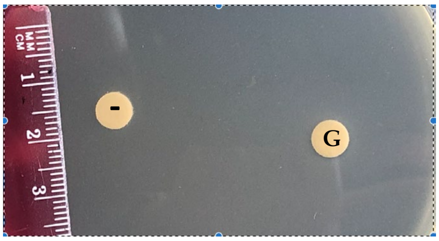

(c)

Figure S1. Disc diffusion method using (**a**) *Escherichia coli* K12 DSM498; (**b**) *Staphylococcus aureus* COL MRSA and (**c**) *Streptococcus mutans* DSM20523. The *S. mutans* strain used in this work displays

resistance to 10 μg of gentamicin. Discs impregnated with bi-distilled water (-) and gentamicin (10 μg) (G).
